# Beyond mitotane: next-generation therapeutic landscapes in adrenocortical carcinoma

**DOI:** 10.1530/EO-26-0040

**Published:** 2026-09-07

**Authors:** Keya Kotecha, Katia Mariniello, Leonardo Guasti

**Affiliations:** Centre for Endocrinology, William Harvey Research Institute, Faculty of Medicine and Dentistry, Queen Mary University of London, London, UK

**Keywords:** immunotherapy, adrenal cortex, carcinoma

## Abstract

The clinical management of adrenocortical carcinoma (ACC) has long been defined by its resistance to conventional therapies. Since the FIRM-ACT trial established the combination of etoposide, doxorubicin, and cisplatin plus mitotane (EDP-M) as the frontline standard, overall survival for metastatic patients has remained stubbornly low. This plateau in efficacy with cytotoxic regimes has necessitated a shift towards precision oncology and immunotherapy. However, translating the success of immune checkpoint inhibitors seen in other malignancies to ACC has proven exceptionally difficult. The primary challenge in the present therapeutic landscape of immunotherapy is the unique biology of the adrenal cortex, which creates an intrinsic immunologically privileged site. This review will address these current immunological hurdles but also explore novel strategies designed to bypass this barrier entirely. By shifting focus towards mechanisms that do not solely rely on the traditional engagement of adaptive immune system – such as the targeted delivery of cytotoxic payloads via antibody–drug conjugates (ADCs) or the androgen-mediated activation of myeloid cells to clear pro-tumoural populations – the field is moving towards a future where the ‘cold’ nature of the tumour microenvironment may no longer dictate the limits of therapeutic success.

## Current hurdles: the barrier of glucocorticoid excess

While the FIRM-ACT regimen remains the gold standard (1), its high toxicity and the requirement for mitotane – a drug with a narrow therapeutic window and significant metabolic side effects – have created an urgent need for alternatives. In current clinical practice, gemcitabine-based combinations, such as gemcitabine plus capecitabine or metronomic 5-fluorouracil, are frequently employed as second-line or salvage therapies, albeit with modest response rates (2, 3). Numerous clinical efforts targeting the IGF and mTOR pathways, alongside the evaluation of radiopharmaceuticals and cyclin-dependent kinase inhibitors, have been reviewed elsewhere (4).

The primary obstacle in contemporary adrenocortical carcinoma (ACC) management remains the tumour’s capacity to stay immunologically invisible. A defining feature of the ACC is the high prevalence of functional, hormone-secreting tumours. Glucocorticoid (GC) excess is the most common functional presentation of ACC and is observed in approximately 50–60% of hormonally active tumours, driving an immunosuppressive tumour microenvironment (5). Both endogenous and synthetic GCs suppress the activity of tumour-infiltrating lymphocytes (TILs), prevent the maturation of antigen-presenting cells, and inhibit the production of pro-inflammatory cytokines. Indeed, a favourable association between the density of CD3^+^ (total T cell pool), CD3^+^CD4^+^ (helper T cells), and CD3^+^CD8^+^ (cytotoxic T cells) TILs and both overall and disease-free survival has been described in a large ACC cohort (6). Beyond hormonal suppression, the genomic landscape of ACC contributes to its non-immunogenic phenotype; molecular profiling has demonstrated that ACC comprises distinct subgroups based on gene mutations/alterations of methylation with divergent biological and therapeutic features. The C1A subgroup is associated with higher mutation rates (especially in the Wnt/β-catenin pathway, TP53) and poor prognosis, whereas C1B tumours generally display a lower mutation rate, with miRNA deregulation being a more favourable clinical outcome (7). Interestingly, a recent steroid phenotype stratification study linked these molecular subgroups to distinct immune landscapes, showing that C1A tumours, characterized by steroid excess, were associated with low human leucocyte antigen (HLA) expression and limited T cell infiltration, likely driven by steroid excess and Wnt/β-catenin signalling. In contrast, C1B tumours, characterized by low steroid production, displayed higher HLA expression, increased infiltration by cytotoxic lymphocytes, and upregulation of immune checkpoints, such as PD-1 and CTLA-4 (8). Together, these findings support molecular and immune stratification as a framework for precision therapeutic approaches in ACC, where C1A tumours may require combination strategies aimed at reversing immune exclusion or antibody–drug conjugates (ADCs), while the C1B subgroup may be intrinsically more sensitive to immune checkpoint blockade. Mechanistic insights from melanoma models suggest that activated β-catenin signalling prevents the secretion of the chemokine CCL4, thereby blocking the recruitment of CD103+ dendritic cells. Without these essential antigen-presenting cells, T cells are never primed to infiltrate the tumour microenvironment (9). This mechanism appears highly relevant to ACC, where high β-catenin expression is inversely correlated with the infiltration of nearly all immune cell lineages and associated with significantly lower programmed death-ligand 1 (PD-L1) expression (10). This clinical observation aligns with broader pan-cancer analyses demonstrating that a T cell-inflamed gene expression signature not only is a primary predictor of favourable prognosis (11), but is also inversely correlated with Wnt/β-catenin signalling, which therefore serves as a dominant mechanism of immune exclusion across diverse solid tumours, including ACC (12). Accordingly, these genomic alterations leave the cancer depleted of immune cells required for immune checkpoint inhibitors (ICIs) to be effective.

Emerging therapeutic strategies directly targeting Wnt/β-catenin signalling are also beginning to enter clinical evaluation in ACC. CY-101 (getacatetide), a first-in-class peptide therapeutic, is proposed to inhibit Wnt/β-catenin signalling via activation of the β-catenin destruction complex while simultaneously inducing cancer cell membrane permeabilization and neoantigen release. In the phase I/IIa CICILIA trial, the ACC sub-cohort demonstrated a disease control rate of 50% (3/6) with monotherapy, including two durable responses exceeding 6 months, both occurring in tumours harbouring β-catenin pathway alterations (13). These preliminary findings have led to the ongoing phase II CLARITY trial (ClinicalTrials.gov: NCT04260529**)** and represent one of the first clinical attempts to therapeutically reverse Wnt/β-catenin-mediated immune exclusion and proliferation in this disease.

Furthermore, ACC highly expresses vascular endothelial growth factor (VEGF) and its receptor VEGF-R2, despite paradoxically low yield micro-vessel density observed in some studies (14, 15). In preclinical models, the multi-kinase inhibitor sunitinib was found to exert direct antiproliferative effects and inhibit steroidogenesis in adrenal cell lines, potentially alleviating the immunosuppressive effects of cortisol excess (15). Despite this compelling rationale, monotherapy approaches have largely failed to meet expectations; early efforts to introduce ICIs and tyrosine kinase inhibitors (TKIs) as single agents demonstrated limited and highly variable clinical efficacy (6, 14, 16).

Prospective evaluation of single-agent ICIs in ACC has generally demonstrated modest efficacy. In an initial phase II trial of pembrolizumab in previously treated ACC (ClinicalTrials.gov: NCT02721732), Habra *et al.* initially reported an objective response rate (ORR) of 14% with a non-progression rate at 27 weeks of 36% (17). Updated follow-up from the same cohort by Chahla *et al.* confirmed durable clinical activity, reporting an ORR of 20% and a 27-week non-progression rate of 30%, although responses remained confined to a minority of patients (18). A subsequent multicentre phase II study (ClinicalTrials.gov: NCT02673333) by Raj *et al.* observed a higher ORR of 23%, although the median progression-free survival remained limited (19). Similarly, nivolumab (ClinicalTrials.gov: NCT02720484) and avelumab (ClinicalTrials.gov: NCT01772004) monotherapy demonstrated ORRs of approximately 11 and 6%, respectively (20, 21); however, interpretation of the latter is complicated by the fact that approximately half of patients remained on concomitant mitotane treatment and mitotane plasma concentrations were not systematically recorded. Historically, occasional durable responses to PD-1 blockade appeared enriched in the minority of ACCs harbouring mismatch repair deficiency (dMMR), microsatellite instability-high (MSI-H) status, or high tumour mutational burden (TMB-high) (19). However, recent comprehensive characterization of the dMMR/MSI landscape in ACC by Altieri *et al.* challenges the predictive value of these biomarkers, demonstrating that dMMR often occurs independently of MSI in ACC and does not reliably predict ICI response (22). Moreover, in the updated pembrolizumab study, microsatellite instability status was unavailable, further limiting conclusions regarding biomarker-driven patient selection (18). Consequently, the optimal strategy for biomarker-driven patient selection in ACC remains highly complex and actively debated.

A recent phase II clinical trial (ClinicalTrials.gov: NCT04318730) evaluated the combination of the anti-PD-1 antibody camrelizumab and the anti-VEGFR agent apatinib. This study demonstrated promising clinical activity in advanced ACC patients who had progressed after standard therapy, achieving a notable ORR of 52%. Biomarker analysis revealed that responders exhibited higher baseline peripheral CD8+ T cell abundance and a higher overlap of clonotypes between tumour-infiltrating and circulating T cells. Additionally, higher levels of CXCR3+ CD8+ T cells and lower levels of immunosuppressive CD4+ populations were associated with more favourable outcomes (23). These findings should be interpreted cautiously given the small sample size (*n* = 21) and the single-arm, open-label design of the phase II study. Furthermore, because VEGFR inhibition itself may exert antitumour and steroidogenesis-modulating effects in ACC, the relative contribution of apatinib versus PD-1 blockade remains uncertain. Dedicated controlled studies will therefore be required to clarify the specific mechanisms underlying clinical benefit. These findings suggest that the integration of anti-angiogenic TKIs with ICIs may successfully reprogramme the ACC microenvironment, sensitizing an immunologically non-permissive niche to checkpoint blockade. This synergistic approach is currently being further validated in the prospective Alliance A092204 trial (ClinicalTrials.gov: NCT06900595), which is investigating the combination of the PD-1 inhibitor cemiplimab and the multi-target TKI cabozantinib. Finally, emerging evidence highlights the critical role of extracellular matrix remodelling in simultaneously driving ACC dissemination and regulating the immune landscape of the niche. In this context, targeting fascin actin-bundling protein 1 (FSCN1) may offer a dual-action solution by both inhibiting metastatic spread and reversing local immunosuppression. In The Cancer Genome Atlas (TCGA)-ACC cohort, FSCN1 expression was found to be negatively associated with intratumoural effector T cell signatures (24). Crucially, pharmacological inhibition of FSCN1 with the small-molecule G2-044 reduced the invasive potential of ACC cells *in vitro* and in zebrafish models. Furthermore, this blockade appeared to actively modulate the inflammatory niche, potentially by upregulating NLRP1 inflammasome components (25). G2-044 is currently being evaluated in phase I clinical trials in other solid tumours (ClinicalTrials.gov: NCT05023486 and NCT03199586). Beyond its role in tumour motility, FSCN1 blockade has been found to reinvigorate antitumour immunity in various syngeneic mouse models, even in tumours previously refractory to PD-1 inhibition (26). Mechanistically, this effect was found to be driven by the inhibition of FSCN1 within conventional dendritic cells (cDCs) rather than within tumour cells, with the blockage enhancing both cDCs’ intratumoural abundance and their capacity for antigen uptake. This myeloid-driven reprogramming ultimately triggered a robust influx of activated CD8+ T cells, translating into a significant survival advantage. Since synergy between G2-044 and ICIs has been observed across multiple cancer types (26), this dual-targeting approach represents a compelling candidate for clinical evaluation in ACC.

## Future directions: circumventing the immune-excluded phenotype

As clinical experience with unmodified immune checkpoint blockade continues to mature, the therapeutic focus has steadily broadened. Rather than attempting to resolve the immunosuppressive architecture of the adrenal niche, strategies are increasingly being investigated to treat ACC as a discrete molecular target. By utilizing precision tools, such as antibody–drug conjugates (ADCs) and chimeric antigen receptor (CAR) T cell therapy, the field aims to deliver potent effector functions directly to the malignancy.

### Antibody–drug conjugates

**Figure 1:**
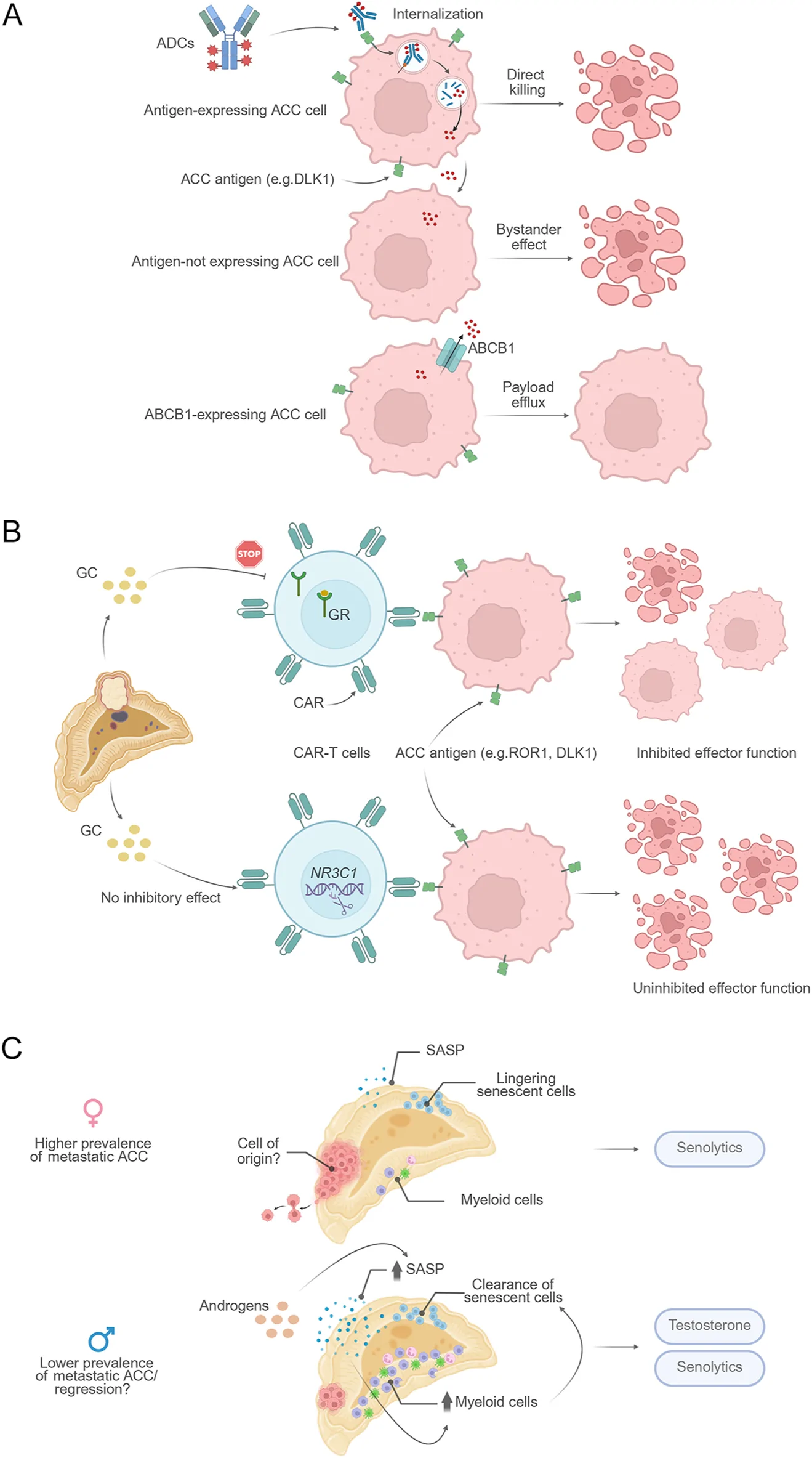
Mechanistic landscape for next-generation therapeutic modalities in ACC. (A) Precision targeting via antibody–drug conjugates (ADCs) bypasses traditional immune exclusion by delivering cytotoxic payloads directly to ACC antigens, such as DLK1. After binding to the antigen on the cell surface, ADCs are internalized via endocytosis and trafficked to the endo-lysosomal compartment. Here, the acidic environment and lysosomal proteases facilitate the proteolytic cleavage or hydrolytic uncoupling of the payload from the antibody, allowing the active drug to be released into the cytosol. When the payload (such as the PBD dimer used in ADCT-701) is hydrophobic (lipophilic) and neutrally charged, it possesses the unique ability to diffuse across the plasma membrane of the primary target cell and enter the surrounding tumour microenvironment. This results in the ‘bystander effect’, where the cytotoxic agent eradicates adjacent tumour cells regardless of their antigen expression. In the context of ACC, this is a significant advantage for two reasons: it allows for the killing of DLK1-negative cells that reside in close proximity to DLK1-positive cells, and the diffused payload can also target the supportive tumour stroma or neovasculature, further destabilizing the malignant niche. Efficacy of certain payloads (such as PBD) may be limited by ABCB1-mediated drug efflux, highly expressed in ACCs. (B) CAR T cell therapy faces significant hurdles in the hormonally active ACC niche, where high GC levels drive T cell exhaustion. CRISPR/Cas9-mediated knockout of the GC receptor (*NR3C1*) may allow these cells to resist steroid-induced suppression and sustain their cytotoxic efficacy *in situ*. (C) Modulation of the innate immune response addresses the sexual dimorphism in myeloid-mediated clearance. While androgens facilitate robust macrophage-led recruitment and phagocytosis of senescent cells in males, an attenuated myeloid response in females allows pre-cancerous populations of still unknown origin to persist. Restoring physiological androgen levels or utilizing senolytics to eliminate these persistent pre-cancerous or pre-cancerous-inducing cell populations may re-engage natural protective mechanisms to eradicate lesions before malignant transformation. Created with Biorender.com.

An ADC functions as a selective molecular delivery vector, consisting of a monoclonal antibody (mAb) (or other mAb-derived formats) covalently linked to a potent cytotoxic payload. This approach could be highly advantageous for ACC because it bypasses the need for T cell recruitment or antigen presentation (Fig. 1A).

The primary target of interest is DLK1 (delta-like non-canonical Notch ligand 1). DLK1 is a paternally expressed transmembrane protein highly active during fetal development with low/nil expression in postnatal tissues (27). Furthermore, DLK1 is aberrantly expressed across cancers of diverse histogenesis, with recent evidence supporting its critical role in the maintenance of aggressive stem cell characteristics and tumour plasticity (28, 29). Using genetic fate mapping and murine ACC models, we have recently identified DLK1 as a *bona fide* marker of a population of adrenocortical stem/progenitor cells. These cells were found to be active during development, near-dormant postnatally, but re-expressed in ACC. Paradoxically, despite its progenitor role, spatial transcriptomic analysis of human ACC samples identified DLK1-expressing cell populations to have increased steroidogenic potential (30). Moreover, DLK1 expression was ubiquitously expressed in a cohort of over 200 human ACC samples and was an independent prognostic marker of recurrence-free survival (30). These findings were independently corroborated by Sun *et al.*, whose broad screening of Notch ligands across the Genotype-Tissue Expression (GTEx) portal and refractory metastatic cancer cohorts identified DLK1 as a uniquely suitable immunotherapeutic target (31). Capitalizing on this high-density surface expression in ACC and in other DLK1-expressing cancers (such as paediatric neuroblastomas and phaeochromocytomas), ADCT-701 has been developed, which is an ADC consisting of a humanized anti-DLK1 mAb conjugated to a pyrrolobenzodiazepine (PBD) dimer payload via a cleavable linker. This payload, once decoupled from the mAb in endo-lysosomal compartments after internalization in DLK1-expressing cells, binds DNA minor groove, creating crosslinks that inhibit replication and transcription and induce cell death. Interestingly, Sun *et al.* showed that, beyond direct cytotoxicity observed in H295R and CU-ACC1 cell lines, ADCT-701 demonstrated ‘bystander killing’, where the intracellularly released PBD, being hydrophobic, can diffuse locally to act on neighbouring DLK1-negative cells (31). Such ‘bystander killing’ is critical for overcoming the mosaic-like expression of DLK1 in ACC, where the intermingling of DLK1-positive and negative cell populations might otherwise allow for small pockets of treatment-resistant clones (30). However, while the ADCT-701 induced complete responses in small cell lung cancer in *in vitro* and *in vivo* models, its efficacy was significantly hindered in a subset of ACC patient-derived xenografts expressing high levels of the drug efflux protein ABCB1/MDR1/P-gp1. Interestingly, DLK1 was found to mediate upregulation of ABCB1 in a NOTCH1-dependent manner. High ABCB1 expression mediated both intrinsic and acquired resistance to the PBD payload, although this resistance could be partially reversed experimentally using ABCB1 inhibitors (31). Currently, ADCT-701 is being evaluated in a phase I dose escalation study for neuroendocrine neoplasms or malignant ACC (ClinicalTrials.gov: NCT06041516). The DLK1–ABCB1 axis suggests that the long-term success of ADCs in ACC may depend on the strategic selection of specific payloads; future strategies should prioritize agents experimentally proven to be poor substrates for the ABCB1 efflux pump, such as the modified anthracycline PNU-159682 (a topoisomerase II inhibitor) or camptothecin derivatives such as exatecan and DXd (topoisomerase I inhibitors) (32).

### CAR-T therapy and the challenge of creating GC-insensitive T cells

Chimeric antigen receptor (CAR) T cell therapy involves genetically engineering a patient’s T-lymphocytes to express synthetic receptors that directly eliminate cells displaying specific surface antigens (Fig. 1B). The phase I LION-1 trial (European Union Clinical Trial number 2024-512019-36-00) is currently evaluating ROR1-specific CAR-T cells in ROR1-expressing cancers, including ACC. ROR1 (receptor tyrosine kinase-like orphan receptor 1) is an oncofetal protein that promotes tumour survival and epithelial-to-mesenchymal transition in cancer stem cells (33) and is highly expressed in ACC with minimal presence in healthy adult tissues, establishing it as a viable target (34, 35). While preliminary data in other malignancies demonstrate safety, the therapeutic impact in solid tumours has remained modest, largely due to poor T cell infiltration (36). As stated, in the ACC microenvironment, high local GC levels exacerbate this challenge by driving the exhaustion and depletion of infiltrating T cells. To counter this, CRISPR/Cas9 is being utilized to knock out the GC receptor (*NR3C1*) within CAR-T products. Preliminary preclinical assessments (currently available as conference proceedings) of human GR-knockout ROR1 CAR-T cells demonstrate that these cells remain functionally unresponsive to GC-mediated immunosuppression, maintaining cytotoxic function even under supraphysiological adrenal steroid concentrations. Furthermore, this preliminary evidence suggests that genetic resistance proves superior to pharmaceutical corticosteroid inhibitors, which inadvertently downregulate ROR1 expression in ACC tumour models (35).

Additionally, DLK1 represents a promising preclinical CAR-T target. While ACC-specific clinical data are currently unavailable, DLK1-specific CAR-T cells have demonstrated potent cytotoxicity, resistance to exhaustion, and significant tumour regression in preclinical hepatocellular carcinoma models, supporting its potential as a viable strategy for refractory solid tumours (37).

### Leveraging myeloid-led clearance of pre-cancerous cells and senescence control

While the ADCs and CAR-T therapies discussed above are actively entering early-phase clinical evaluation, the field is also exploring promising, albeit still speculative, translational strategies still in the preclinical stage. Unlike these adaptive immune-directed strategies, which seek to enhance tumour recognition and elimination through engineered or redirected immune mechanisms, emerging evidence suggests that harnessing the innate immune response may represent a complementary therapeutic pillar. Supporting this concept, recent studies have identified defective macrophage-mediated immune surveillance as a key determinant of ACC progression. An emerging paradigm of ACC pathogenesis centres on the delicate balance between the induction of cellular senescence and the efficiency of macrophage-led clearance; when this process fails, driven by biological sex or ageing, mouse models have shown that tumour progression and metastatic spread can ensue (Fig. 1C).

Two studies have characterized a driver of the sexual dimorphism inherent to ACC, which is disproportionately observed in women. In a mouse model utilizing the adrenal-specific loss of the WNT inhibitor zinc and ring finger 3 (Znrf3, a common driver in ACC), Wilmouth *et al.* observed that cellular senescence occurred in both sexes. However, the subsequent disease trajectory was dictated by the hormonal milieu; in males, androgens facilitated the recruitment of tumouricidal macrophages, which in turn phagocytosed senescent steroidogenic cells, leading to spontaneous hyperplasia regression and prevention of malignancy. Conversely, in females, macrophage recruitment was markedly delayed and attenuated, allowing senescent cells to persist. This persistence in females was associated with a higher incidence of ACC development. This sexual dimorphism was corroborated by human TCGA data, where male ACC samples exhibited enriched signatures for phagocytic macrophages; notably, this immune profile correlated with significantly improved overall survival compared with female cohorts (38).

These findings were reinforced and extended by Warde *et al.* by using the same Znrf3-deficient mouse model. Their work demonstrated that Znrf3*-*driven cellular senescence was activated when adrenocortical cells switched from hyperplasia to regression. Senescence cells were also shown to establish a chronic senescence-associated secretory phenotype (SASP), characterized by the secretion of established SASP components, such as IL1a, IL1b, IL6, INFg, TGFb3, and MMP12. Again, a clear sex-based divergence was observed in how SASP attracted myeloid cells (mostly phagocytic macrophages but also other myeloid cells and some lymphoid lineages), with males showing both an earlier onset and a higher total volume of cell recruitment than females. Because the phenotypic shift was accelerated in males and characterized by a more intense SASP profile, the resulting recruitment of tumouricidal cells was significantly more effective than that observed in females. Like in the previous study, androgens were found to mediate the amplified SASP response in males. Over time, in the female cohort, this SASP-driven remodelling of the adrenal microenvironment transformed the local niche into a permissive pre-malignant environment, fuelling the transition from hyperplasia to aggressive, metastatic carcinoma. These observations were clinically validated in humans by developing a myeloid response score (MRS); when applied to the TCGA-ACC cohort, a low MRS, predominantly found in female patients, correlated with significantly worse progression-free survival (39).

Although the definitive cell of origin for ACC remains to be identified, the findings from these two studies bring the field a step closer by highlighting potential candidates for malignant transformation. These candidates likely include either lingering senescent cells that successfully escape cell cycle arrest while acquiring cancer stem cell characteristics (40) or neighbouring non-senescent populations, such as fully steroidogenic cells, transiently amplifying cells, or progenitor-like cells, that are pathologically altered by the paracrine SASP effects of the surrounding senescent niche. Even in the absence of a definitive cell of origin, the discovery of a sex-dimorphic, androgen-dependent clearance of potential pre-cancerous or cancer-inducing cells in the adrenal cortex provides a hypothesis-generating framework for exploring malignant transformation. In theory, maintaining physiological testosterone levels could help preserve the innate immune system’s capacity to clear senescent, pro-tumorigenic cells. However, this concept remains strictly speculative. The current mechanistic evidence relies on a single *Znrf3*-deficient mouse model, and the supporting human myeloid response score (MRS) data are correlative. Furthermore, because approximately 30% of ACCs are androgen-secreting and a subset is clinically androgen-driven, any potential clinical hypothesis regarding androgen restoration should be restricted to non-androgen-secreting cases. In these specific patients, androgen levels are frequently suppressed, either through cortisol-induced inhibition of the hypothalamic–pituitary–gonadal axis or as a side effect of mitotane therapy (41, 42). Furthermore, the discovery that persistent senescent cells might drive metastatic progression positions senolytic agents as a potent new therapeutic modality (43). Ultimately, shifting focus towards myeloid-modulating and senescence-targeting strategies could offer a biologically tailored approach, leveraging innate protective mechanisms to eradicate lesions before malignant transformation.

## Conclusions and future horizons

The therapeutic landscape of ACC remains a challenging clinical frontier, long constrained by the limitations of conventional cytotoxic regimens and the intrinsic immunologically privileged nature of the adrenal niche. While traditional immune checkpoint inhibition has yielded modest single-agent success, the principal unmet need rests in overcoming GC- and Wnt/β-catenin-mediated immune exclusion. Addressing this requires a strategic shift towards novel research priorities, specifically the deployment of precision vectors such as antibody–drug conjugates, genetic engineering to render CAR-T cells steroid-insensitive, and the exploration of innate myeloid-led or senolytic interventions. Ultimately, advancing these next-generation modalities beyond the preclinical arena in such a rare and aggressive malignancy demands a concerted shift towards international collaboration and adaptive platform trials designed to rapidly validate biomarker-stratified precision therapies.

## Declaration of interest

The authors declare that there is no conflict of interest that could be perceived as prejudicing the impartiality of the work reported.

## Funding

This work was supported by grants from the Nefkens Adrenal Cancer Foundation, the UK Medical Research Council, and Barts Charity.

## References

[1] Fassnacht M, Terzolo M, Allolio B, et al. Combination chemotherapy in advanced adrenocortical carcinoma. N Engl J Med 2012 366 2189–2197. (10.1056/nejmoa1200966) 22551107

[2] Sperone P, Ferrero A, Daffara F, et al. Gemcitabine plus metronomic 5-fluorouracil or capecitabine as a second-/third-line chemotherapy in advanced adrenocortical carcinoma: a multicenter phase II study. Endocr Relat Cancer 2010 17 445–453. (10.1677/erc-09-0281) 20410174

[3] Henning JEK, Deutschbein T, Altieri B, et al. Gemcitabine-based chemotherapy in adrenocortical carcinoma: a multicenter study of efficacy and predictive factors. J Clin Endocrinol Metab 2017 102 4323–4332. (10.1210/jc.2017-01624) 29092062

[4] Altieri B, Ronchi CL, Kroiss M, et al. Next-generation therapies for adrenocortical carcinoma. Best Pract Res Clin Endocrinol Metab 2020 34 101434. (10.1016/j.beem.2020.101434) 32622829

[5] Fassnacht M, Dekkers OM, Else T, et al. European Society of Endocrinology Clinical Practice Guidelines on the management of adrenocortical carcinoma in adults, in collaboration with the European Network for the Study of Adrenal Tumors. Eur J Endocrinol 2018 179 G1–G46. (10.1530/eje-18-0608) 30299884

[6] Landwehr LS, Altieri B, Schreiner J, et al. Interplay between glucocorticoids and tumor-infiltrating lymphocytes on the prognosis of adrenocortical carcinoma. J Immunother Cancer 2020 8 e000469. (10.1136/jitc-2019-000469) PMC7264832 32474412

[7] Assie G, Letouze E, Fassnacht M, et al. Integrated genomic characterization of adrenocortical carcinoma. Nat Genet 2014 46 607–612. (10.1038/ng.2953) 24747642

[8] Giner IS, Resende JSS, Muzzi JCD, et al. Steroid phenotype stratification reveals distinct HLA expression signatures in adrenocortical carcinoma. Cancers 2026 18 229. (10.3390/cancers18020229) PMC12839242 41595150

[9] Spranger S, Bao R & Gajewski TF. Melanoma-intrinsic beta-catenin signalling prevents anti-tumour immunity. Nature 2015 523 231–235. (10.1038/nature14404) 25970248

[10] Liu S, Ding G, Zhou Z, et al. Beta-Catenin-driven adrenocortical carcinoma is characterized with immune exclusion. Onco Targets Ther 2018 11 2029–2036. (10.2147/ott.s159979) PMC5898592 29670378

[11] Ott PA, Bang YJ, Piha-Paul SA, et al. T-Cell-Inflamed gene-expression profile, programmed death ligand 1 expression, and tumor mutational burden predict efficacy in patients treated with pembrolizumab across 20 cancers: KEYNOTE-028. J Clin Oncol 2019 37 318–327. (10.1200/jco.2018.78.2276) 30557521

[12] Luke JJ, Bao R, Sweis RF, et al. WNT/beta-catenin pathway activation correlates with immune exclusion across human cancers. Clin Cancer Res 2019 25 3074–3083. (10.1158/1078-0432.ccr-18-1942) PMC6522301 30635339

[13] Sikkema BJ, Champiat S, Gort EH, et al. 661P Safety and activity of CY-101 in patients with advanced solid tumors: the phase I/IIa CICILIA trial. Ann Oncol 2024 35(Supp. 2) S520–S521. (10.1016/j.annonc.2024.08.727)

[14] Pereira SS, Oliveira S, Monteiro MP, et al. Angiogenesis in the normal adrenal fetal cortex and adrenocortical tumors. Cancers 2021 13 1030. (10.3390/cancers13051030) PMC7957756 33804534

[15] Kroiss M, Reuss M, Kuhner D, et al. Sunitinib inhibits cell proliferation and alters steroidogenesis by down-regulation of HSD3B2 in adrenocortical carcinoma cells. Front Endocrinol 2011 2 27. (10.3389/fendo.2011.00027) PMC3356136 22654799

[16] Flauto F & Damiano V. The efficacy and safety of multi-kinase inhibitors in adrenocortical carcinoma: a systematic review and single-arm meta-analysis. Cancers 2025 17 2004. (10.3390/cancers17122004) PMC12190831 40563655

[17] Habra MA, Stephen B, Campbell M, et al. Phase II clinical trial of pembrolizumab efficacy and safety in advanced adrenocortical carcinoma. J Immunother Cancer 2019 7 253. (10.1186/s40425-019-0722-x) PMC6751592 31533818

[18] Chahla B, Stephen B, Song J, et al. Phase 2 study of monotherapy with pembrolizumab for advanced adrenocortical carcinoma. J Immunother Precis Oncol 2025 8 242–248. (10.36401/JIPO-25-6) PMC12549218 41143139

[19] Raj N, Zheng Y, Kelly V, et al. PD-1 blockade in advanced adrenocortical carcinoma. J Clin Oncol 2020 38 71–80. (10.1200/jco.19.01586) PMC7351334 31644329

[20] Carneiro BA, Konda B, Costa RB, et al. Nivolumab in metastatic adrenocortical carcinoma: results of a phase 2 trial. J Clin Endocrinol Metab 2019 104 6193–6200. (10.1210/jc.2019-00600) 31276163

[21] Le Tourneau C, Hoimes C, Zarwan C, et al. Avelumab in patients with previously treated metastatic adrenocortical carcinoma: phase 1b results from the JAVELIN solid tumor trial. J Immunother Cancer 2018 6 111. (10.1186/s40425-018-0424-9) PMC6198369 30348224

[22] Altieri B, Kircher S, Herterich S, et al. Mismatch repair deficiency and microsatellite instability in adrenocortical carcinoma. ESMO Open 2026 11 106030. (10.1016/j.esmoop.2025.106030) PMC12906187 41650745

[23] Zhu YC, Wei ZG, Wang JJ, et al. Camrelizumab plus apatinib for previously treated advanced adrenocortical carcinoma: a single-arm phase 2 trial. Nat Commun 2024 15 10371. (10.1038/s41467-024-54661-9) PMC11604670 39609453

[24] Liang J, Liu Z, Wei X, et al. Expression of FSCN1 and FOXM1 are associated with poor prognosis of adrenocortical carcinoma patients. BMC Cancer 2019 19 1165. (10.1186/s12885-019-6389-3) PMC6884893 31783819

[25] Ruggiero C, Tamburello M, Rossini E, et al. FSCN1 as a new druggable target in adrenocortical carcinoma. Int J Cancer 2023 153 210–223. (10.1002/ijc.34526) 36971100

[26] Wang Y, Song M, Liu M, et al. Fascin inhibitor increases intratumoral dendritic cell activation and anti-cancer immunity. Cell Rep 2021 35 108948. (10.1016/j.celrep.2021.108948) PMC8050791 33826900

[27] Traustadottir GA, Lagoni LV, Ankerstjerne LBS, et al. The imprinted gene Delta like non-canonical Notch ligand 1 (Dlk1) is conserved in mammals, and serves a growth modulatory role during tissue development and regeneration through Notch dependent and independent mechanisms. Cytokine Growth Factor Rev 2019 46 17–27. (10.1016/j.cytogfr.2019.03.006) 30930082

[28] Pittaway JFH, Lipsos C, Mariniello K, et al. The role of delta-like non-canonical Notch ligand 1 (DLK1) in cancer. Endocr Relat Cancer 2021 28 R271–R287. (10.1530/erc-21-0208) 34627131

[29] Grassi ES & Pietras A. Emerging roles of DLK1 in the stem cell niche and cancer stemness. J Histochem Cytochem 2022 70 17–28. (10.1369/00221554211048951) PMC8721543 34606325

[30] Mariniello K, Pittaway JFH, Altieri B, et al. Dlk1 is a novel adrenocortical stem/progenitor cell marker that predicts malignancy in adrenocortical carcinoma. Cancer Commun (Lond) 2025 45 663–668. (10.1002/cac2.70012) PMC12187577 40035383

[31] Sun NY, Kumar S, Kim YS, et al. Identification of the Notch ligand DLK1 as an immunotherapeutic target and regulator of tumor cell plasticity and chemoresistance in adrenocortical carcinoma. Nat Commun 2025 16 5511. (10.1038/s41467-025-60649-w) PMC12216638 40595495

[32] Roth JS, Guo H, Chen L, et al. Identification of antibody-drug conjugate payloads that are substrates of ATP-binding cassette drug efflux transporters. Cancer Drug Resist 2026 9 2. (10.20517/cdr.2025.151) PMC12883344 41668804

[33] Jung MS, Choi WY, Zhang W, et al. The role of receptor tyrosine kinase-like orphan receptor 1 (ROR1) in cancer stem cell signaling. Int J Mol Sci 2025 26 7828. (10.3390/ijms26167828) PMC12386924 40869147

[34] Zhang S, Chen L, Wang-Rodriguez J, et al. The onco-embryonic antigen ROR1 is expressed by a variety of human cancers. Am J Pathol 2012 181 1903–1910. (10.1016/j.ajpath.2012.08.024) PMC3509760 23041612

[35] Schauer M, Altieri B, Redondo-Frutos R, et al. ROR-1 specific CAR-T cells with CRISPR/CAS9 mediated glucocorticoid receptor-knockout exert potent antitumor efficacy in advanced adrenocortical carcinoma. Endocr Abstr 2023 93 OC20. (10.1530/endoabs.93.OC20)

[36] Jaeger-Ruckstuhl CA, Specht JM, Voutsinas JM, et al. Phase I study of ROR1-Specific CAR-T cells in advanced hematopoietic and epithelial malignancies. Clin Cancer Res 2025 31 503–514. (10.1158/1078-0432.ccr-24-2172) PMC11788652 39466024

[37] Zhai Y, He K, Huang L, et al. DLK1-directed chimeric antigen receptor T-cell therapy for hepatocellular carcinoma. Liver Int 2022 42 2524–2537. (10.1111/liv.15411) 36002393

[38] Wilmouth JJ Jr, Olabe J, Garcia-Garcia D, et al. Sexually dimorphic activation of innate antitumor immunity prevents adrenocortical carcinoma development. Sci Adv 2022 8 eadd0422. (10.1126/sciadv.add0422) PMC9565812 36240276

[39] Warde KM, Smith LJ, Liu L, et al. Senescence-induced immune remodeling facilitates metastatic adrenal cancer in a sex-dimorphic manner. Nat Aging 2023 3 846–865. (10.1038/s43587-023-00420-2) PMC11534150 37231196

[40] Milanovic M, Fan DNY, Belenki D, et al. Senescence-associated reprogramming promotes cancer stemness. Nature 2018 553 96–100. (10.1038/nature25167) 29258294

[41] Canu L, Sparano C, Naletto L, et al. Hypogonadism and sexual function in men affected by adrenocortical carcinoma under mitotane therapy. Front Endocrinol 2023 14 1320722. (10.3389/fendo.2023.1320722) PMC10807287 38269251

[42] Delbarba A, Cosentini D, Facondo P, et al. Androgen serum levels in male patients with adrenocortical carcinoma given mitotane therapy: a single center retrospective longitudinal study. Front Endocrinol 2023 14 1128061. (10.3389/fendo.2023.1128061) PMC10108714 37077359

[43] Meguro S & Nakanishi M. Cellular senescence in the cancer microenvironment. J Biochem 2025 177 171–176. (10.1093/jb/mvaf001) 39760850

